# From Negentropy to Norms – A Gain-Based Theory of Agency: Kerdotism

**DOI:** 10.1007/s12124-026-10001-8

**Published:** 2026-05-01

**Authors:** Michael Kypreos

**Affiliations:** University of Heidelberg, Heidelberg, Germany

**Keywords:** Agency, Normativity, Action theory, Teleology, Homeostasis, Negentropy

## Abstract

This paper proposes a vertically integrated framework of agency that connects thermodynamic constraints on living systems with agent-level evaluation and higher-order normative phenomena. Starting from the constraint that biological systems must maintain their organization under entropic pressure, we introduce a Principle of Teleological Necessity that models action as systematically oriented toward an antecedent end. On this basis, we develop an evaluative calculus that treats value and norm-guided behavior in terms of perceived gain and loss. The framework distinguishes five axiological dimensions—from somatic homeostasis to altruistic resonance—and specifies how inhomogeneous trade-offs can be represented within a common evaluative medium. We introduce epistemic transduction, together with a principle of proximity, to model how subjective perspective and local informational constraints shape deliberation and choice. Finally, we show how patterns recognizable as morality and possession can arise as stabilizations in networks of interacting agents, and we indicate points of contact with empirical work in action theory, moral psychology, and complex systems science.

## Introduction

A central challenge in contemporary philosophy of mind and science is to explain how simple, thermodynamically constrained systems give rise to complex, value-laden forms of organization. Despite important advances in systems biology, there is no clear consensus on a vertically integrated account of this transition. In particular, existing accounts do not yet show how thermodynamic constraints on living systems culminate, in a continuous and principled way, in evaluative structures such as actions, goals, and norms.

In the absence of such an account, our theoretical landscape remains fragmented: there is as yet no broadly adopted calculus that unifies thermodynamic primitives with higher-order evaluative constructs while making an agent’s deliberative architecture explicit. Many current models struggle to characterize the transition from metabolic self-maintenance to agency that is plausibly described as normative, and they do not specify how normative guidance is realized at the level of deliberation. Attempts to bridge this gap often rely on proxy measures (e.g., informational or energetic quantities) or permissive uses of key terms, making it difficult to locate the transition to norm-guided agency within a single agent-level architecture. This gap reflects a mismatch between the quantities available at the thermodynamic level and the agent-level categories required to model evaluation, deliberation, and action-guiding normativity. Models that place explanatory weight on a small set of global quantities can leave underdetermined how heterogeneous evaluative considerations are integrated within an agent, limiting their usefulness as accounts of deliberative structure.

Arguably the most prominent of such models is the Free Energy Principle proposed by Friston (2010). It posits that, to maintain their organization and persist over time, self-organizing systems must minimize an upper bound on “variational free energy”, often glossed as an information-theoretic proxy related to surprisal. While mathematically well-defined, this framework has been debated with respect to how directly it yields discriminative, empirically testable predictions at biologically realistic levels of description, and how its key quantities should be operationalized in concrete models.

Turning to another model, the enactive approach conceives cognition as embodied action, implying that living systems are autonomous insofar as they actively generate the conditions necessary for their own survival (Maturana & Varela, 1980; Thompson, 2007; Varela et al., 1992). However, this approach leaves contested the status of representations, and it does not articulate the transition from mere viability to norm-guided agency without stipulating normativity at the outset. As a result, the agent’s internal deliberative architecture can remain under-specified.

Another prominent naturalistic description of normativity is provided through teleosemantics (Millikan, 1984; Neander, 1991; Papineau, 1987). It defines normative concepts such as right or wrong by proper function. Normativity is therefore understood in an etiological manner; hence, normative values are defined by what they evolved to be. Simultaneously, this approach shifts normative gravity onto evolutionary history, leaving an explanatory gap regarding the agent’s internal deliberative architecture. Consequently, teleofunctional normativity is often too coarse-grained to serve as a commensurable and scalable structure of value.

This paper proposes an axiomatized framework that links thermodynamic constraints on persistence to an agent-level evaluative architecture. The core commitment is a constitutive thesis about action: the principle of teleological necessity, according to which action is essentially purposive, and merely stochastic behavior does not qualify as action in the relevant sense. Building on this, we introduce strict definitions that shift explanatory load from abstract evaluative labels (“good”/“bad”) to a more tractable gain–loss format, understood as transitions toward or away from systemic optima. The resulting framework makes heterogeneous considerations commensurable within a single deliberative architecture and thereby represents cross-domain trade-offs explicitly. We then propose two further principles that shape deliberation—proximity (discounting by epistemic distance) and homeostasis (dynamic reweighting by deprivation/satiation)—and use them to model how stable, norm-like strategies can arise in populations of interacting agents. The distinctive contribution lies in making the intra-agent weighing procedure explicit in a commensurable format.

The framework does not derive first-order moral truths; it offers a unifying conceptual construct and a set of constraints that can interface with empirical work in decision theory, action theory, and moral psychology. Normativity is treated in a reasons-first and broadly constitutivist register: the practical *should* is fixed relative to an agent’s constitutive aim and its registered reasons, rather than by mind-independent moral facts.

This constitutivist commitment is not offered as a semantic analysis of moral predicates, nor as a defense of mind-independent moral realism. Instead, it fixes the explanatory target as practical normativity within an agent-level architecture: the question is how reasons are registered, rendered commensurable, and made action-guiding for systems constrained by persistence.

Section “Background and Problem Setting” states the problem and desiderata; Section “Theoretical Core” develops the axioms and the gain–loss architecture. Section “Normativity and Evaluation: Target and Payoff” clarifies the target notion of normativity and the payoff of the framework; Section “Objections and Replies” addresses central objections; Section “Comparison with Leading Accounts” situates the view relative to leading accounts. Section “Methodological Approach” briefly summarizes the methodological construction, and Section “Conclusion” concludes.

## Background and Problem Setting

### Problem Statement

Contemporary models of action and agency have not yet provided an explicit account of intra-agent evaluative architecture that is grounded in constraints on systemic persistence under entropic pressure while scaling to macroscopic normative categories in social and deliberative contexts. In particular, highly abstract normative concepts such as morality and possession remain poorly linked to empirical primitives and therefore lack principled constraints. The result is a fragmentary picture of evaluation: existing accounts do not specify how heterogeneous factors are integrated within an agent and how norm-guidance is realized in the evaluative process. What is needed is a principled account that makes the evaluative architecture explicit in a commensurable format, so that cross-domain trade-offs and the stabilization of norm-guided behavior can be modeled within a single agent-level framework.

### Background and Motivation

Bridging the gap between thermodynamic primitives, agency, and normativity is a complex undertaking, as it requires an explicit cross-scale unity that many frameworks do not model. This transition also requires a principled connection between diverse phenomenological evidence and a single basal primitive. Securing this unity via proxy metrics that are not themselves part of the evaluative process can yield an account that becomes unstable and drifts into arbitrariness. A successful framework therefore requires a common empirical anchor. Here, agency is limited to living organisms; artificial agents are excluded insofar as the argument targets physical constraints and phenomenological access in biological systems. Such systems are uniformly subject to entropic pressure over time; hence entropy and the associated constraints provide a common empirical basis.

However, even when a model is anchored in entropy, the agent’s internal mechanisms remain poorly defined. Evaluation integrates heterogeneous factors, and without an explicit architecture, their commensurability remains underdetermined, leaving integration unconstrained and inviting arbitrariness. This is particularly salient when connecting concepts such as morality and possession to empirical primitives, since the link is not transparent. Having now identified these bottlenecks, we examine contemporary approaches with respect to whether they (I) ground agency in entropy, (II) specify an intra-agent evaluative architecture, (III) render factors commensurable within the agent, and (IV) explain macroscopic normative categories without proxy substitution.

### Related Approaches and Outstanding Gaps

We now evaluate prominent naturalistic approaches to action and agency with respect to four questions: (I) whether they provide an explicit grounding in entropic constraints on persistence, (II) whether they specify an intra-agent evaluative architecture, (III) whether they render heterogeneous considerations commensurable within the agent, and (IV) whether they account for macroscopic normative categories without substituting proxy metrics for evaluative structure. The discussion focuses on three influential proposals already flagged in the Introduction: the Free Energy Principle (Friston, 2010), enactivism (Maturana & Varela, 1980; Thompson, 2007; Varela et al., 1992) and teleosemantics (Millikan, 1984; Neander, 1991; Papineau, 1987).

The Free Energy Principle proposed by Friston (2010) holds that self-organizing systems must minimize variational free energy in order to maintain homeostasis. In this way, variational free energy functions as a naturalistic anchor point: it links action to persistence under entropic pressure, though the connection to entropy is mediated by information-theoretic quantities rather than stated in purely thermodynamic terms. Variational free energy can be understood, at a high level, as a variational bound related to surprisal (i.e., negative log evidence) and thus as an information-theoretic proxy for the system’s expected discrepancy between its generative model and incoming observations. Building upon this central construct, the model proposes a unified formalism, linking perception–action–learning with systemic persistence.

(I) The model offers an anchor point that is connected to entropy as a concept via information-theoretic quantities and axiomatic grounding, but this link requires empirical specification and scaling. (II) The intra-agent evaluative architecture is based upon a priori expectations of minimized free energy (e.g., prior preferences over outcomes), where what counts as “good” is fixed by expected outcomes shaped by learning and model specification. The normative weight of the model is consequently partially disaggregated into model specifications. (III) Though the model presents free energy as a commensurable medium, commensurability is often achieved through the choice of priors, preferences, and model structure, rather than through a sophisticated intra-agent weighting architecture (Bruineberg et al., 2018). (IV) While some moral and social phenomena can be modeled, concepts such as norms, morals, and value can thereby enter as encoded expectations rather than emerging from the model’s structure alone. The Free Energy Principle represents a case where a highly general, albeit for specific applications very effective, proxy objective is used and preferences as well as higher-order normative constructs have to be made explicit, since the intra-agent architecture is not sufficiently defined for them to straightforwardly emerge (Andrews, 2021).

The enactive approach associated with (Thompson, 2007; Varela et al., 1992) holds that cognition is fundamentally embodied and enacted in action, and that autonomy consists in the organism’s ongoing self-maintenance through which it generates and sustains the conditions of its own viability (Di Paolo, 2005). Enactivism typically focuses on dynamic agent-environment-couplings instead of assuming internal representations. In this sense, meaning and value emerge through the relations of the agent with its environment instead of through arbitrary utility scalars. Similarly, norms are typically explained via agent interaction and coordination.

(I) This model offers a weaker empirical anchor point, emphasizing cognition and interaction within the environment rather than a unifying metric. (II) Enactivism takes a skeptical stance toward agent-internal representations and, therefore, does not motivate a sophisticated intra-agent evaluative architecture. (III) Consequently, commensurability is not established via a common internal medium, leaving the relevant intra-agent weighing structure implicit and difficult to operationalize (Barandiaran et al., 2009). (IV) The model posits that core normative concepts such as value and norms emerge through the agent’s interaction with its environment; however, the internal architecture underwriting this interaction remains largely unspecified, limiting mechanistic explanatory traction. By externalizing normativity into relational dynamics, the approach circumvents the need for an explicit intra-agent weighing architecture; yet, absent a clear metric, such descriptions drift into arbitrariness, and their analytical and predictive scope remains constrained.

Teleosemantics, associated with Millikan (1984, 1989); Neander (1991), explains agentic states and mechanisms in terms of evolutionary adaptation and proper function. This introduces correctness conditions (i.e., correct/incorrect) into the model, allowing a notion of normativity to emerge without invoking moral concepts. Misrepresentation is explained via malfunction or by tokening a mechanism outside the conditions for which it was selected. Actions are also postulated to be correct in relation to their aims, where these aims are set by the functions of the mechanisms.

(I) Teleosemantics offers a naturalistic anchor point within evolutionary biology, grounding representational content in selection history and proper function (Wright, 1973). However, this anchor is primarily etiological and historical and therefore does not straightforwardly provide a basal, cross-scale metric in the way an entropy-based constraint might. This shifts much explanatory weight onto function-ascriptions and historical accounts, which can be underdetermined in concrete cases. (II) Instead of specifying an agent-internal evaluative architecture, teleosemantics focuses on the correctness conditions of representations. This provides basal building blocks through which a limited notion of normativity (correct/incorrect) can emerge; however, the account remains largely at the level of proper function rather than an explicit intra-agent weighing process. (III) Teleosemantics does not offer a commensurable medium that an evaluative architecture could weigh. Instead, it explains when representations and actions count as correct relative to their functions, leaving commensurability effectively externalized into functional roles and historical explanations. (IV) Teleosemantics helps to explain stable contents as representations within a population; however, the jump from functional normativity to moral normativity typically requires further substantive assumptions. As the approach does not itself provide a detailed intra-agent evaluative architecture, its predictive grip on norm-guided behavior can remain limited without additional modeling commitments.

Having now examined the three most closely related approaches, it becomes clear that each leaves at least one of the desiderata only partially addressed. Accordingly, the central aim—to establish a conceptual framework that unites an explicit agent architecture with entropy as an empirical anchor while scaling to emergent normative concepts and preserving intra-agent commensurability—remains unmet.

### Desiderata for a Naturalistic Account of Normativity

Having now examined contemporary accounts of action theory, we can state desiderata for a naturalistic account of normativity. The foregoing discussion motivates the following criteria:

1. **Empirical anchoring in persistence under entropic pressure.** The account must fix a non-arbitrary basal constraint on agency by tying evaluation to the conditions under which living systems remain viable (cf. Section “Background and Motivation”).
2. **An explicit intra-agent evaluative architecture.** The model must specify (rather than merely presuppose) the internal structure by which an agent registers, compares, and weights heterogeneous considerations in deliberation.
3. **Intra-agent commensurability.** The account must provide a commensurable format within the agent that allows heterogeneous factors to be integrated without shifting the normative burden into ad hoc proxy metrics (e.g., a gain–loss format that can be uniformly applied across domains).
4. **A decision-relevant metric that goes beyond mere stochasticity minimization.** While low stochasticity (or related proxy objectives) functions as a persistence-relevant factor, it must not be the sole metric of evaluation; the model must articulate how action-guidance is generated from a decision-relevant internal metric.
5. **Temporal structure and feedback.** The architecture must explain how evaluation unfolds across time and how outcomes update future weighting via an intra-agent feedback loop.
6. **Cross-scale continuity to macroscopic normative categories.** The account must show how higher-order normative phenomena (e.g., moral evaluation and possession) can be derived from, or constrained by, the intra-agent architecture and its empirical anchor, rather than being imported via external assumptions.

With the desiderata now established, we can shift our focus to the scope and commitments.

### Scope, Commitments and Limitations

The scope of Kerdotism, as developed in this work, is delimited by the present aim. Although Kerdotistic resources may be extended to further higher-order concepts, the present discussion focuses on (I) making the deliberative architecture explicit and (II) deriving an account of moral evaluation and possession. Accordingly, the analysis is primarily intra-agent and small-scale: the framework is developed at maximal granularity at the level of an individual evaluator. This restriction is methodological rather than substantive; the proposed architecture applies across contexts, from everyday action to extreme situations, insofar as the relevant evaluative structure is in place. Where the framework appeals to phenomenological evidence, the relevant agents are assumed to possess conscious access to gain–loss registration.

As discussed above, the model employs persistence under entropic pressure as its empirical anchor. This anchor functions as a constraint on living systems, and thereby fixes the background problem to which action and deliberation are responsive. The entropic grounding itself does not yield norms; rather, it provides a basal condition under which norm-guided behavior must be stabilized. A central commitment of Kerdotism is therefore the postulation of an internal evaluative architecture. Although current scientific methods do not permit a direct mechanistic description of this architecture, it is introduced as a theoretically tractable locus of evaluation that permits a systematic connection between persistence constraints and moral phenomena. On this basis, the gain–loss format is adopted as a commensurable, idealized metric for representing heterogeneous evaluative inputs. In describing the model as naturalistic, the claim is constructive and explanatory, not reductive in the sense of offering a complete empirical implementation.

The framework is not empirical in the sense of providing an implementable control architecture; instead, it is constrained by empirical concepts and guided by phenomenological evidence about evaluative experience and offers some empirical predictions. A corresponding limitation is under-specification: in particular, the notion of preemptive expected gain requires further formal and empirical refinement. Moreover, the model is limited in scope insofar as it provides an agent-level conceptual framework rather than a full social theory.

These restrictions also clarify the intended use cases. Even prior to full formalization, Kerdotism can be applied analytically to model intra-agent trade-offs in gain–loss terms and to track how such trade-offs constrain higher-order normative categories.

## Theoretical Core

### The Principle of Teleological Necessity

To connect persistence under entropic pressure to deliberation, we begin from a constraint that is comparatively stable across agents: living systems must continually regulate their own organization in order to retain systemic integrity under entropic pressure. For macroscopic organisms in open, fluctuating environments, such regulation is not exhausted by passive thermodynamic self-organization, but requires behaviorally mediated control over organism–environment exchanges. This motivates a constitutive constraint on what counts as *action* in the sense relevant to agency and evaluation.

#### Definition 1

(Principle of Teleological Necessity) An *action* can only be conceived (in the sense relevant to agency and evaluation) insofar as it is guided by an antecedent end, i.e., only insofar as it is selectable as a means in the service of maintaining or improving the agent’s regulated integrity.

Here “regulated integrity” is a placeholder for whatever variables the agent registers as viability-relevant in its own control architecture (to be made explicit in the gain–loss format introduced below). The principle fixes which episodes are in scope as *actions*; it does not yet specify the decision rule by which an agent selects among candidate actions.

The rationale is selection-theoretic and cost-based. Maintaining macroscopic organization requires continual throughput of resources and the avoidance of catastrophic loss. Accordingly, behavioral dispositions that systematically consume resources without contributing to viability are evolutionarily unstable: they impose uncompensated energetic and opportunity costs and will tend to be eliminated or suppressed by selection and developmental control. Conversely, what remains stable at organismic time-scales is end-directed, viability-tracking guidance: action selection that is organized around anticipated contributions to regulated integrity.

The principle is therefore not intended as a merely verbal constraint, nor does it deny that organisms exhibit seemingly random behavior. Rather, it holds that any *action* is purposive for the agent, even when that purpose is not transparent from an external point of view; behavior that appears random to an observer may still be end-directed relative to the agent’s own regulatory constraints. In this respect, the principle is an axiomatically stated counterpart to familiar thermodynamic motivations in the background literature on life as “feeding on negentropy” (Schrödinger, 1944), and it is compatible with accounts that formulate persistence as minimizing long-run surprisal or related quantities (see, e.g., Friston 2010). Within the present framework, it functions as the foundational principle: guided action is treated as a necessity for systemic persistence of macroscopic organisms and therefore constrains action to a necessarily purposive undertaking.

### The Basal Definitions

Tying directly into the principle of teleological necessity, the biological substrate allows a corresponding treatment of the binary evaluative categories *good* and *bad*. This paper uses these predicates in an operational sense: as tracking labels for intra-agent representations, not as mind-independent properties. The present framework adopts the following basal identification. This is a modeling move: it shifts the definitional weight from highly abstract predicates to a gain–loss vocabulary that is more tractable for representing deliberation.

#### Definition 2

(Basal identifications) In the present framework, *good* is the way gain is perceived and *bad* is the way loss is perceived. Accordingly, *gain* is defined as any *perceived* enhancement of an individual’s state, and *loss* as a *perceived* regression of that state, relative to a reference state.

Gain and loss are defined at the level of epistemic registration and may diverge from objective utility outcomes; in this sense, objective utility functions as an external success condition rather than as part of the definitional base of the calculus.

These definitions remain parsimonious while being anchored in a reference state, i.e., a baseline relative to which a transition can count as enhancement or regression for the agent. In this way, the framework connects subjective perception with objective description: the agent’s evaluative registration is subjective, yet it is structured by the notion of transitions relative to a state that functions as a reference point. On this basis, gain and loss can be treated as commensurable, enabling a categorization that remains in direct contact with lived experience, and thereby provides phenomenological grounding for the conceptual scheme.

The bridge to practical normativity is built by treating deliberation as the internal integration of persistence-relevant constraints under subjectively registered information. On this picture, reasons enter the architecture as registered gain–loss considerations that can be weighed against each other only if they are rendered commensurable in a single internal medium. The gain–loss format is introduced as precisely that medium: it provides the minimal architecture needed to represent trade-offs among heterogeneous consequences and thereby to make the agent-relative *should* action-guiding rather than merely stipulative.

However, to achieve a commensurable basis in a non-arbitrary manner, the heterogeneous conglomerate subsumed under gain and loss must be analytically disaggregated until elementary aspects remain that can be classified as positive or negative in the basal sense just defined, i.e., as registered enhancements or regressions relative to the reference state. The framework therefore proceeds reductively, while imposing a clear stopping condition in order to avoid spiraling into arbitrariness. The resulting elementary aspects constitute the units that are ultimately weighed in the evaluation once they have been axiologically refined. In this sense, an atomistic deconstruction of gain and loss, followed by an assessment of the resulting elementary aspects, is intended to mirror the functional organization of conscious evaluative pathways.

This conceptual architecture forms the crude corpus of the abstract calculation. Since empirical observation of the relevant internal processes is currently beyond our scientific limitations, the construction is offered as an *a priori* model constrained by internal consistency and guided by subjective access to evaluative experience in living organisms. The result is a form of phenomenological isomorphism: the framework aims to provide a conceptual mirror of the qualitative weighing of internal states.

### The Taxonomy of Gain

To maintain oversight, the model systematizes gain and loss by categorizing them with respect to the environment in which a decision is made. Here, the environment of an action comprises what is affected by that action in ways that can be registered by the agent, since only such factors can enter into the agent’s evaluation of the action and its consequences. On this basis, the taxonomy distinguishes two overarching domains. The first comprises gain and loss that are limited to the individual and its non-conscious environment; the second comprises gain and loss that arise within a conscious environment. This separation is introduced to capture the role of conscious observation in the evaluative registration of gain and loss. For example, a suboptimal motion in a non-conscious environment constitutes loss in the form of metabolic expenditure; in a conscious environment, a further loss can arise insofar as the agent is subject to evaluative observation by other agents. To operationalize this distinction, gain is disaggregated into five constitutive dimensions. The domain distinction is introduced as a coarse-grained motivation for why conscious environments introduce additional evaluative registers; the five dimensions then provide the operative disaggregation of gain and loss and can cut across this domain boundary. The dimensions are not assumed to be mutually exclusive: a single action may yield gain and loss components across multiple dimensions. While the five dimensions are introduced as constitutive categories, their primary role is classificatory: they function as a compression scheme that keeps heterogeneous gain–loss aspects surveyable. Accordingly, in applications one can treat all elementary aspects within a given dimension as sharing a single homeostatic scaling factor, thereby trading granularity for tractability.

#### The First Dimension

The first dimension concerns the intrasubjective domain. It includes direct bodily stimuli (e.g., nociceptive registration) as well as the immediate somatic consequences the agent undergoes in interaction with other agents. Such consequences may be physical (e.g., bodily harm) or affective (e.g., mental distress). The first dimension therefore comprises gain and loss as experienced by the agent with respect to its somatic substrate. Since it includes the vital aspects of systemic persistence—in line with Damasio’s somatic marker hypothesis (Damasio, 1994)—it renders the first dimension the agent’s baseline priority within the present taxonomy.

#### The Second Dimension

Moving beyond the somatic substrate, the second dimension concerns the asocial mediation of utility. It comprises epistemically registered gain and loss that arise through the instrumental exploitation of a non-conscious environment, and it is grounded in the acquisition and use of abiotic tools or non-sentient biota. Paradigm cases include tool use in complex organisms and practices such as fermentation, which leverage microbial processing to obtain nutrient-dense substrates and stabilize exogenous resources. Within this dimension, changes of state are registered insofar as the environment is instrumentalized into utility-bearing structures, thereby facilitating and augmenting the agent’s actions and increasing its teleological reach.

#### The Third Dimension

The third dimension concerns gain and loss that are epistemically registered through interaction with and evaluation by other conscious and intentional agents. Paradigm cases include reputational gains acquired through the performance of specific behaviors under the evaluative gaze of a social audience. To maintain analytical rigor, the taxonomy distinguishes the causal origin of a stimulus from its constitutive domain. This distinction applies throughout the taxonomy. For instance, interaction with a social audience can trigger affective distress; insofar as this distress is a somatic state that can occur independently of intersubjective constitution, it is classified under the first dimension. By contrast, relational devaluation is classified under the third dimension, since it depends on the presence of a social referent and consists of a shift in how the agent is evaluated by others. Within this dimension, changes in state reflect alterations in external evaluation, and therefore amount to gain or loss in intersubjective leverage, affecting the influence of the agent beyond purely instrumental means.

#### The Fourth Dimension

The fourth dimension concerns gain and loss epistemically registered through collective synergy, i.e., through actions that result in mutual utility for the agent and other peers as opposed to the reputational utility characteristic of the third dimension and the exclusively other-directed utility characteristic of the fifth dimension. The relevant peers may be directly known or only registered by the agent as a collective entity. Gain in this dimension is emergent in the sense that it shifts the focus of evaluation from aspects affecting only the agent to a collectively registered gain shared by the agent and its peers. In small groups, this dimension is often co-instantiated with the third dimension, since an action may simultaneously yield reputational gain and mutual utility; communal sharing of resources among proximal peers provides a simple illustration. In larger groups, fourth-dimensional gain can become the overriding evaluative weight, motivating actions with terminal individual costs in order to realize collective gain. Although this dimension exhibits affinities with both the third and the fifth dimensions, it is treated here as axiologically distinct from each; in particular, fourth-dimensional gain can remain grounded in shared outcomes, whereas fifth-dimensional gain involves the assimilation of the other’s state as such and third-dimensional gain need not track the other’s gain as such.

#### The Fifth Dimension

The fifth dimension concerns gain and loss epistemically registered through acts of altruism (fifth-dimensional gain), i.e., actions that provide exclusive utility to other agents. Within the present framework, such acts are explained in terms of an epistemic assimilation of the other’s state into the agent’s own evaluation, yielding an abstract form of gain that is not reducible to reputational incentives, strategic leverage, or direct mutual benefit. Gain in this dimension is dissociated from the agent’s own functional utility; instead, it is predicated on evaluative isomorphism between individuals. This evaluative isomorphism—empirically supported by the discovery of mirror mechanisms (Rizzolatti & Sinigaglia, 2008)—enables the agent to register the other’s internal state transitions with reduced epistemic opacity, thereby aligning the other’s epistemic registration of gain and loss more closely with the agent’s own evaluation. Accordingly, evaluative isomorphism functions as a limiting factor of altruistic action by constraining experienced commensurability between agents. The background of altruistic action will be expanded upon as the scope of the framework increases. In what follows, the reference state invoked in the basal definitions is treated as fixed by the agent’s regulated integrity profile and its homeostatic baselines across these dimensions.

### The Corporeal Substrate

Having now established an empirical grounding and a taxonomy, while circumventing definitional arbitrariness through strict definitions of *good* and *bad*, the transition from taxonomy to decision theory requires a clear description of the weighting parameters that determine the axiological gravity of the dimensions. These parameters permit analytical use of the model by establishing commensurability between the five dimensions of gain, rendering them constitutive for the framework’s operational applicability. The first basal postulate—distinct from the principle of teleological necessity—concerns the subjective nature of epistemic registration. The agent can only epistemically perceive its environment through its senses, followed by the transduction of stimuli into internal representations; this is what we refer to as *registration*, and it is the basis on which the agent can weigh and include inputs in an evaluation.

#### Definition 3

(First basal postulate (Principle of Subjectivity)) All information about an agent’s environment available to the agent is relative to its own epistemic access; accordingly, unregistered features are functionally inert for the agent’s evaluation, even if they matter for objective outcomes. Moreover, the interpretation of registered stimuli is itself subjective, so epistemic perception and transduction need not coincide with the objective state of affairs.

As established, registration is subjective, and interpretations and conceptual understandings of the environment differ between individuals, yielding an irreducible degree of incommensurability through subjectivity alone; this incommensurability is inter-agentic (i.e., it constrains comparison across agents), not a denial that a single agent can render its own registered inputs commensurable for deliberation. At the same time, epistemic registration is subject to representational distortion of ontological reality. In a broadly Kantian sense (Kant, 1781), registration can be treated as a synthetic unity of heterogeneous inputs. To maintain analytical clarity, we adopt the following definition.

#### Definition 4

(Epistemic registration) Epistemic registration is the integrated internal representation of the environment produced by epistemic perception of stimuli and epistemic transduction, i.e., the processing by which such stimuli are conceptualized and rendered comparable for evaluation.

While perception offers the agent physical details of its environment, captured through its sensory organs, transduction establishes the inputs as concepts, incorporating them into a context that allows for commensurability between inputs.

As the agent can only evaluate what it registers, anything unregistered is functionally inconsequential to the evaluation, though it may lead to objective utility deficits. Such limitations established by subjectivity can be illustrated through a case analysis. This does not defend a global metaphysical determinism; it fixes a conditional claim: given a fixed epistemic registration, evaluative output is determined by the agent’s internal mapping. In this sense, “determinism” is an architectural thesis about the evaluation-to-action mapping within the model, not a metaphysical claim about the world at large. Consider two fully isomorphic agents—genetically identical, sharing identical ontogenetic histories, and registering the same situation identically—who are placed in a forced-choice paradigm. Under these stipulated conditions, the agents would necessarily maintain behavioral isomorphism; any other outcome would require an extra variable absent from the initial parameters, given the principle of subjectivity. Unless full isomorphism is attained, the analysis and understanding of another agent’s actions is therefore strictly delimited by the subjectivity of epistemic registration. Any framework utilizing subjective observation cannot achieve predictive certainty due to inherent individuality; it can only approach the isomorphic ideal.

This thought experiment also motivates nomological determinism for the framework as a whole, in the sense that every action is the inevitable output of the agent’s internal logic. As an illustration of how far structurally constrained, mechanistic models can go in generating predictive leverage, contemporary large-scale connectome-based brain simulations already yield non-arbitrary sensorimotor predictions at the level of whole circuits (Shiu et al., 2024). Moreover, recent studies suggest that such connectome-scale simulations can be executed as explicit mechanistic models on dedicated hardware, supporting the feasibility of deterministic rule-governed large-scale neural dynamics (Wang et al., 2025). While these results do not by themselves establish metaphysical determinism, they help motivate the present architectural reading of determinism as a claim about constrained evaluation-to-action mappings.

Returning to the model, determinism arises at the level of evaluation: conditional on a fixed epistemic registration, action is the necessary output of the agent’s internal mapping. However, strict determinism appears to lead to a teleological impasse in cases of absolute equipollence, as shown in the thought experiment of Buridan’s Ass: an ass between two identical haybales at the exact distance would starve if both options are registered identically and no other factor forces motion. A structurally analogous point can be made using large language models as a heuristic comparison: absent an external probabilistic seed, their outputs are deterministic relative to fixed inputs (Wolfram, 2023). Given that a conscious agent would not behave as the Buridanic thought experiment predicts, the external probabilistic seed must have been stripped away by the stipulation of absolute equipollence. Yet, within the present framework, the seed can be located in the main limiting factor of our observation, namely subjectivity. Because the agent’s epistemic register is inextricably bound to an inherently indeterminate environment, any interaction introduces an external probabilistic seed, and divergence in registration is not merely probable but nomologically necessary, since epistemic interaction is contingent upon probabilistic fluctuations of the environment.

This intrinsic transductive asymmetry negates the possibility of identical registration, rendering an epistemic agent ontologically immune to a Buridanic deadlock. Any observation of supposed stochasticity in an internal state transition is therefore effectively indistinguishable from the stochasticity of the observed environment. Applying Ockham’s Razor, the postulation of intrinsic neurological noise becomes theoretically redundant. If such noise were to exist, it would be functionally isomorphic to the external probabilistic seed; the cognitive evaluative process can thus be modeled as nomologically rigid while remaining indifferent to the specific causal origin of the stochastic variance. This does not constitute a proven fact but a methodological modeling move.

### The Second Basal Postulate

Building upon the nomological rigidity established above, the second basal postulate fixes the direction of the evaluative process.

#### Definition 5

(Second basal postulate) Conditional on a given epistemic registration, an agent selects the action that it evaluates as yielding the greatest projected net gain.

This is treated as an operational expression of the negentropic drive discussed earlier. Within the present framework, *gain* is the registered index of negentropic success. Gain and loss are defined at the level of epistemic registration and need not coincide with objective utility outcomes. Negentropic success is not mere energy conservation or biological survival; it denotes the maintenance of organized control—the governance of the agent’s state against entropic noise. *Loss* represents a depreciation of the agent’s state, yielding non-convertible attrition. A *negentropic failure* is thus an action whose registered outcome involves greater loss than gain. Therefore, the negentropic drive is operationalized as the imperative to convert energy into gain. Consuming energy for a zero-utility conversion, or for a conversion that depreciates the agent’s state, is treated (for present modeling purposes) as purposeless action. Such action is classified as negentropic failure.^1^

### The Principle of Proximity

To quantify gain and loss, it is crucial to isolate the constitutive variables through which the agent can weigh elementary aspects against each other. In analyzing behavior, a clear fundamental principle emerges: the principle of proximity.

#### Definition 6

(Principle of Proximity) The principle of proximity acts as an epistemic filter, weighing anticipated outcomes (gain and loss) based on their immediacy to the agent. This entails a devaluation of registered magnitudes as epistemic distance to the agentic horizon increases.

This principle takes shape as hyperbolic discounting (Ainslie, 2001), which is exemplified by the common paradigm of immediate loss outweighing distant gain even when the gain is objectively greater than the loss—a pattern classically observed in spatiotemporal contexts. However, the definition must be extended to include statistical proximity. Within this framework, a prospective consequence is subject to axiological attenuation insofar as it is registered as a low-probability event. Analogously to spatial or temporal distance, statistical distance reduces the registered magnitude of a consequence, as the agent prioritizes causal certainty over stochastic risks. Importantly, this attenuation describes a default weighting tendency; it can be counteracted or even inverted when salient internal representations compress epistemic distance. Consequently, evaluation is not dependent on objective distance, but on epistemic proximity. Intensive cognitive engagement or vivid representation can reduce the distance perceived by the agent, thus altering the axiological significance of a distant event. This helps explain risk-taking behavior such as gambling as a distortion in epistemic registration: internal simulation renders prospective gain salient, inflating its registered probability. The resulting virtual axiological inflation can induce enactments that culminate in loss, even when the agent is explicitly aware of unfavorable objective odds.

This is a consequence of the agent’s negentropic drive. As a projected outcome increases in registered distance for the evaluative agent, it becomes less certain to be registered due to stochastic interference. Here interference is understood as exogenous—arising from the environment and the causal fragility of extended action–outcome chains—rather than as intrinsic indeterminacy in the evaluative process. The principle therefore acts as a filter against such interference by preferring immediate certain gain over distant uncertain gain, reducing the variance of expected gain by minimizing causal fragility. Concurrently, it acts as a constraint, precluding the agent from allocating resources across dimensions toward highly uncertain causal pathways at the cost of immediate degradation of the somatic state. Ultimately, it reduces the variance of the system’s future by maximizing the probability of systemic continuity in the present, retaining the somatic state as the ontological prerequisite for future action. This depreciating effect is functionally symmetric and applies to distant loss as well, enabling actions that yield smaller immediate gain while deferring a greater loss. This results in temporal myopia, manifested as structural vulnerability in observable agentic behavior. In this sense, proximity is a stability-oriented principle that incurs predictable myopic costs as a trade-off.

### The Principle of Homeostasis

As discussed above, the inherent negentropic drive motivates a system of priority within an agent, i.e., a weighting regime over epistemically registered consequences, of which the principle of proximity constitutes one independent scaling variable. Another such variable is captured by a quasi-hierarchical tiering of evaluative dimensions. As established previously, the five dimensions of gain and loss are analytically distinct categories under which the consequences of an action can be subsumed. These dimensions exhibit architectural layering: the first dimension encompasses somatic gain and loss and thereby forms the basal condition of systemic persistence, whereas the fifth dimension concerns the most abstract and strictly delimited gain and loss associated with altruistic action. The basal role of the first dimension gives it priority with respect to the maintenance of the agent’s somatic state. This does not entail that first-dimensional gain or loss always outweighs gain or loss in other dimensions. However, it does imply that somatic needs are weighed by their necessity for substrate integrity since they enable systemic continuity.

Consequently, the evaluative weight of gain or loss in a given dimension is dynamically modulated by the homeostatic deviation of the agent’s state in that dimension. Here, *homeostatic deviation* refers to deviation from a neutral state, not only in the somatic dimension but across all dimensions. For example, an abundance of physical resources can depreciate both the prospective gain and the prospective loss associated with the second dimension, insofar as the agent’s integrity is not directly threatened. In this sense, homeostasis provides an operational baseline that marks the dynamic threshold of need, where abundance transitions into insufficiency.

#### Definition 7

(Principle of Homeostasis) An agent adjusts the evaluative weight of epistemically registered gain and loss in proportion to the magnitude of homeostatic deviation in the relevant dimension.

The resulting utility profile is typically asymmetrical and is consistent with the loss aversion/protective bias formalized by Kahneman and Tversky (1979): survival-critical loss-avoidance is prioritized over satiation-induced indifference. This correspondence functions as an empirical signature, not a derivation. The principle of homeostasis thereby entails the quasi-hierarchical structure noted above. The utility weighting profile of the first dimension is the steepest due to its importance for systemic integrity, whereas the other dimensions exhibit progressively attenuated weighting gradients as error tolerance increases with diminishing immediate impact on systemic integrity. In other words, homeostatic weighting operationalizes the negentropic drive by directing evaluative pressure toward deviations that threaten substrate integrity, while permitting comparatively greater tolerance where deviations are non-critical. The principle of homeostasis therefore constitutes another independent scaling variable that impacts the evaluative weight of gain and loss.

### The Third Basal Postulate

However, to achieve commensurability between the dimensions of gain and loss within a single agent’s deliberation, a further postulate is required.

#### Definition 8

(Third basal postulate) Any registered gain (and loss), regardless of dimension, is functionally isomorphic in the sense that it can be represented in a homogeneous gain–loss format, differing only in magnitude. The gain–loss magnitude thereby functions as the commensurable medium for cross-dimensional trade-offs.

This is an intra-agent commensurability claim and is compatible with inter-agent differences in registration. It is motivated by a minimal constraint on deliberation: unless heterogeneous considerations are commensurable in a common internal medium, no genuine cross-domain comparison or trade-off could occur—yet agents manifestly do compare and arbitrate among heterogeneous prospects. In this respect, the postulate is not an additional normative premise but a constraint on what it is to model deliberation as trade-off-sensitive at all. This postulate ties into epistemic transduction. Because registered inputs are processed into internal representations that can be conceptualized and compared, the framework treats transduction as yielding a homogeneous format in which heterogeneous considerations can be weighed. Accordingly, transduction targets precisely the features of epistemic registration that bear on the agent’s regulated integrity state; features that do not bear on that state are functionally inert for evaluation. Since the gain–loss format is agent-relative, transduction does not discard information; it translates each elementary aspect into the deliberation-relevant variable it contributes, namely its predicted gain–loss magnitude. The computational burden is thus shifted to magnitude assignment, preserving parsimony without narrowing the framework’s analytical scope.

Having already identified proximity and homeostasis as factors that modulate magnitude, we treat them as acting on the axiological substrate—that is, the magnitude-assignment structure operative in preemptive evaluation—to determine the expected gain and loss associated with an action. The axiological substrate is fixed in the preemptive evaluation of a possible action and depends on the a priori magnitude of its anticipated consequences, i.e., the significance of the change of state predicted by the agent on the basis of its epistemic registration. The evaluative calculation preceding action is therefore based on expected gain and loss magnitudes, taking into account both proximity and homeostasis as factors. To connect evaluation to enactment, the framework introduces a threshold condition.

#### Definition 9

(Action threshold) The *action threshold* is the minimum margin by which an action must be evaluated as net-positive in order to be enacted. An agent enacts an action only if its expected gain not only exceeds its expected loss but exceeds it sufficiently to compensate for stochastic uncertainty in the action’s consequences.

Phenomenologically, this threshold is not strict; near the tipping point it becomes uncertain, yielding temporary impasse when an action is evaluated as only marginally net-positive. This reflects stochasticity in the agent’s interactions with an unpredictable medium. Minimal gain may fail to compensate for probabilistic variance in consequences, rendering execution risk too high relative to prospective reward.

This threshold-uncertainty is distinct from the principle of proximity. Proximity is already accounted for within the evaluation; the residual uncertainty concerns the chance of failure even given a net-positive assessment. When faced with such an impasse, the agent returns to the evaluative apparatus, attempting to incorporate further aspects of its epistemic registration or to reinterpret existing perceptions, thereby recalibrating the evaluation and extending deliberation. Such indecisiveness cannot be sustained indefinitely, since deliberation incurs systemic cost and opportunity loss while latent gain remains unrealized.

#### Decision Procedure (pseudocode)

To make the foregoing architecture operationally explicit, the following pseudocode summarizes the preemptive evaluation that precedes enactment. It is intentionally schematic: it fixes the order of computation, while leaving open how magnitudes are estimated in concrete empirical models.

**Algorithm 1:**
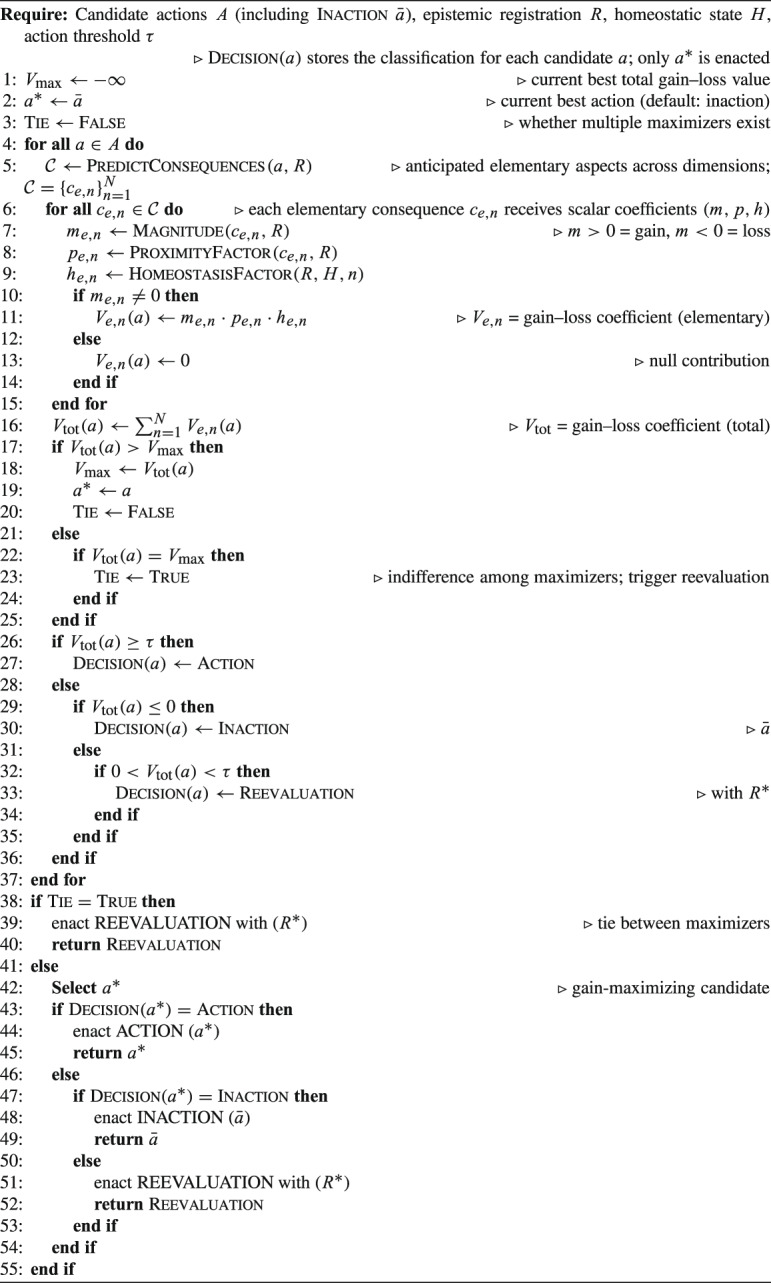
Preemptive evaluation and action selection (schematic).

### The Malleability of the Cognitive Architecture

Having now established the mechanism by which the agent decides on an action, the reciprocal relation of the agent and the action necessitates further examination. In particular, any adequate agent-level account must allow that evaluative dispositions can be revised in light of registered outcomes. Revisiting the thought experiment of the two identical agents grants further insight into the nature of this relation. By establishing that two identical agents would perform isomorphic actions when faced with an identical epistemic registration of a situation, the thought experiment fixes a baseline of behavioral isomorphism. Comparing this to the phenomenological evidence of individuality between agents in reality, two causal determinants of individuality can be distinguished: genetic background and lived experience. If we hold fixed only genetic identity while decoupling lived experience, the agents’ actions would lose the previous isomorphism. Conversely, if we differentiate genetic background while holding fixed lived experience, the same loss of isomorphism follows. By specifically retaining the factor of identical epistemic registration, stochasticity in registration as well as differing registration due to non-identical experiences is rendered impossible; this isolates the evaluative mapping—the assignment of gain–loss magnitudes to registered inputs—as the remaining functional locus of divergence under the stipulated conditions.

Hence, the evaluative mapping itself must have lost the previous isomorphism through the differing factors. What follows most directly is that the mapping from epistemically registered inputs to gain–loss magnitudes, as well as the estimation of proximity or homeostasis, is plastic: it can be recalibrated by experience. While such recalibration could in principle occur solely at the level of weighting within a fixed representational scheme, it is also plausible that learning affects which aspects of a situation are treated as salient or evidential for evaluation. In that weaker and operational sense, differences in genetic background and lived experience can shape both (I) evaluative weighting and (II) the feature-extraction procedures that make consequences commensurable for the agent. Accordingly, the framework treats the cognitive architecture as dynamic and adaptive, changing how the agent registers and evaluates a situation based on previous evaluative outcomes.

This kind of recalibration is suggested by familiar cases in which prior experiences reshape what is registered as threatening or safe, even in environments lacking objective threats (as discussed, for example, in connection with trauma-related generalization; see the systematic review in Dymond et al. 2015). Examining epistemic perception further reveals that the agent’s epistemic reach is not limited to low-level physical sensory input; rather, affective appraisals can be understood as higher-level outputs of transduction that translate complex social and relational patterns into evaluatively usable signals. Therefore, emotions provide a compressed, action-guiding input for evaluation that results through abstract interactions, enabling the agent to act in a manner that will result in gain in a conscious environment. In this sense, emotions as a sensory medium reflect higher-order transductive processing, making it possible for an agent to directly link its action to higher-dimensional consequences in a conscious environment even though such consequences are not straightforwardly readable off purely physical descriptors. This is exemplified by an agonistic facial expression: while physically merely a contraction of certain facial muscles, it is registered via an affective metric so as to carry evaluative weight. This can mitigate uncertainty by providing the agent with epistemic leverage on the likely intentions of other agents, allowing it to dynamically adjust the action accordingly.

The mechanism leading to this inherent malleability of the cognitive architecture through the accumulation of different experiences can be further explored by examining the concept of an action. As established previously, the agent’s epistemic registration, its evaluation, and the resulting action are all based on subjectivity. This subjectivity results in an inherent uncertainty about the consequences following the action. Yet, as the agent can only register the consequences after the action has been carried out, the preceding evaluation of an action cannot incorporate the experiences resulting from the action into the agent’s evaluative apparatus. Therefore, a successive evaluation of the action based on the epistemic registration of the consequences is necessary. Similarly, because an action can be altered during enactment based upon epistemic registration of environmental change, this also necessitates a concurrent evaluation. This entails that a continuous state of evaluation takes place within the agent that can be categorized as preceding evaluation, concurrent evaluation, and successive evaluation. An important detail in this description is that inaction represents a state of inhibitory control. As inaction can lead to gain and loss and also constitutes a mode of negentropic guidance, it must be a valid output of evaluation and teleologically equivalent to an action. Inaction therefore often reactively secures the current homeostasis of the agent by refraining from an action that may interfere with the status quo.

Shifting the focus back to the continuous nature of evaluation, the evaluation of an action is subject to change if the epistemic registration of the environment changes. This change may occur during any evaluative phase, yet it can only affect the present action during the preceding and concurrent phases of evaluation. Instead, a change in evaluation in the successive phase leads to a feedback loop, shaping the cognitive architecture through the epistemic registration of consequences. The extent of dynamic adaptation increases proportionally to the variance between the predicted result and the registered consequence. This deviation may be positive or negative. However, negative deviation, similarly to a negative change in state discussed in the principle of homeostasis, generally leads to greater and more fundamental changes. Once more, this is due to the potential for total systemic failure when the consequences differ greatly from the predicted result in a negative direction, while a great deviation in a positive direction can only lead to a homeostatic enhancement. In this sense, a system may unexpectedly improve greatly many times, yet it may only suffer a catastrophic failure once. Avoidance of an unexpected negative consequence therefore takes priority over the maximization of positive consequences. The evaluation therefore consists of a perpetual and progressive feedback loop, shaping the agent to best utilize its environment based on the experiences it has previously had. This dynamic shaping of the cognitive architecture is illustrated by the paradigm of classical conditioning (Pavlov, 1927). Here, the successive evaluation of a neutral stimulus in combination with somatic gain results in a recalibration of the transductive apparatus. This effectively elevates a stimulus, previously registered as neutral, to a process of axiological significance. Such a change is described to be achievable through both appetitive and aversive conditioning. The latter is often found to be more effective, with evidence that acquisition learning is stronger for aversive than for appetitive events (Schaaf et al., 2022). This is consistent with the framework’s prediction of a protective bias and with the progressive feedback-loop picture. Additionally, it supports the polar classification of systemic changes into the categories of positive and negative, while leaving room for the possibility that apparently neutral cues can remain informationally relevant (e.g., by shaping expectations) even when they are not themselves assigned a non-zero gain–loss magnitude.

### The Derivation of Collective Behavior

Having established the malleability of the cognitive substrate, we can conclude the theoretical core by extending the intra-agent feedback loop to inter-agent dynamics, and thereby to morals. On an empirical construal, morals are emergent: they arise as regularities in how agents adapt their evaluative mapping in response to registered gain and loss in repeated interaction.

Morals primarily concern interaction among conscious agents because the conscious environment differs structurally from the non-conscious environment. Whereas the non-conscious environment acts non-strategically and without intention, other agents pursue their own gain in intentional and comparatively unpredictable ways, and thus constitute a concentrated source of both prospective loss and prospective gain for systemic integrity. Consequently, agentic interaction is not merely facilitative but can become constitutive of persistence (e.g., through coordination, coalition formation, and mutual protection).

This social setting also provides a behavioral basis for fifth-dimensional (altruistic) gain. In altruistic acts, the agent does not typically receive classical gain in return at the time of acting; nevertheless, it may register the act as net-positive. Even where such registration is not grounded in an explicit expectation of return, reciprocal altruism (Trivers, 1971) can stabilize altruistic policies at the population level by increasing the likelihood of aid in future states of need. This stabilizing role of reciprocity must be distinguished from the fifth-dimensional gain of altruism itself. The latter consists of an internal abstract gain produced by epistemic assimilation of the other’s state into the agent’s own evaluation, and can therefore remain present even when future reciprocation is uncertain.

The commensurability between individuals functions here as a contra-stochastic metric: by reducing epistemic opacity, it correlates with the perceived reliability of the other’s action when needed.

At first glance, altruistic action may appear to conflict with the principle of proximity, which attenuates distant and low-probability consequences and would, absent further mechanisms, tend to exclude costly action without immediate classical return. However, within the present framework, proximity is a weighting variable rather than a categorical exclusion. In cases of sufficiently high commensurability, the other’s gain and loss can be registered with reduced epistemic opacity and elevated salience, allowing the agent to experience non-classical altruistic gain in the present and thereby to act without coupling the motivation to the expectation of reciprocation. Over repeated interaction, policies that elicit reciprocity will in expectation increase the stability of the involved agents’ states, which in turn explains why such a pattern of registration and action can be adaptively stabilized. Commensurability simultaneously acts as a limiter, since an unconstrained altruistic drive would risk systemic failure.

Against this background, morals are treated as the population-level stabilization of strategies that tend, in expectation, to yield maximal gain and minimal loss in social interaction (Axelrod, 1984; Bicchieri, 2006; Skyrms, 1996). A simple thought experiment illustrates the mechanism. If an agent adapted to an entirely non-conscious environment is introduced into a social setting, it will initially prioritize only first- and second-dimensional gain. If this yields third- and fourth-dimensional loss (e.g., via retaliation, exclusion, or reputational devaluation), such loss enters successive evaluation and progressively recalibrates the mapping, thereby enforcing preexisting social regularities without requiring direct instruction.

By extension, this yields the basis for possession. Stable interaction requires clear boundaries regarding access to material resources; such boundaries are stabilized when they reduce conflict and enforcement costs by correlating control with predictable enforcement capacities (protective and, where applicable, retaliatory) (Bicchieri, 2006). In this sense, possession is not a primitive relation but an emergent, socially stabilized constraint on material interaction.

Within the present framework, the basic problem is not to explain how an agent comes to stand in a mysterious dyadic relation to an object, but to explain how a population of agents converges on and maintains practical constraints on access and use. Possession is therefore treated as a boundary-setting regularity: it fixes which agents are permitted to treat a resource as instrumentally available (second-dimensional gain) without incurring compensating third- and fourth-dimensional loss through predictable social responses.

This also clarifies the sense in which possession differs from mere physical control. A transient episode of control can occur without any stable constraint on others; by contrast, possession in the present sense requires that the boundary be socially legible and sufficiently stable across interaction for it to enter agents’ preemptive evaluation as an expected cost structure. When these expectations stabilize, agents treat violations as loss-relevant, and the resulting threat of retaliation, exclusion, or reputational devaluation becomes part of the ordinary incentive landscape that keeps access patterns stable over time. Possession is thus analyzed as a constraint that is sustained by interaction, rather than as a fundamental property of objects.

Although moral salience is typically highest in the conscious environment, moral standards can be extended to non-conscious entities insofar as they are linked to agentic gain and loss (e.g., environmental care as it bears on the long-run integrity of populations of agents). Moreover, moralization can occur through agentification^2^: when an agent misattributes commensurability to a non-conscious entity, it may register that entity under social-style constraints and form bonds that recruit moral evaluation (Dennett, 1987). This supports the present framework’s central claim: morals are not a dissociated objective medium, but an empirically constrained, dynamically shifting mesh of gain–loss adaptations across interacting agents.

### Case Study: Capitalism

Taking the application of Kerdotism further, we treat capitalism as a case study for how the foregoing architecture yields concrete predictions about exchange behavior that can be assessed against the literature. Capitalism is a phenomenon that aggregates human action collectively into a productive force via the commensurable medium of currency. However, while commensurable, it is not identical in value between agents. This follows from the principle of homeostasis. Currency acts as a non-conscious object falling under the second dimension of gain, and therefore attains value through its uses. An insufficient amount of currency within the possession of the agent endangers its systemic integrity by indirectly threatening loss of the first dimension, effectively scaling the value according to the principle of homeostasis alongside the affected factors such as hunger (Shah et al., 2012). When an overabundance of currency is present, the value drops according to the principle of homeostasis, as much more marginal forms of short-term gain (i.e., large impulse purchases) present a higher gain to the agent than saving the currency would (Ahl et al., 2023).

The following sketch applies the foregoing architecture to exchange-interactions. While the value of the currency fluctuates, the architecture through which it is converted remains rigid. Examining an exchange of currency for goods reveals that every agent involved will typically favor personal gain (Dimensions 1–4) over the other agent’s gain. This is a consequence of the principle of proximity, the principle of homeostasis, and the subjectivity of the agent. Therefore, the agent must register what it is acquiring as having higher value than what it exchanges for it.^3^ Similarly, the other agent involved will register that which it receives to be more valuable than that which it exchanges. If both agents were to register both objects identically, no exchange could ever take place, thereby underscoring the role of subjectivity within the agent.

Even though the exchange discussed is a simplified model that assumes the interaction takes place within a non-conscious environment, it closely describes modern-day acts of exchange. As production is increasingly outsourced and monopolized, and as online-shopping takes over larger parts of the market, exchange-interactions increasingly move toward this model.

Since online shopping does not involve direct human interaction, it is dissociated from the consciousness behind the digital storefront, resulting in agents interacting with online stores as they would interact with their non-conscious environment. In the vocabulary of social commerce, this dissociation is described as reduced online presence: the interaction cues that would ordinarily sustain an evaluative gaze are attenuated, and the exchange is thereby processed in a more impersonal, instrumentally mediated format (Gefen & Straub, 2004; Hassanein & Head, 2007).

This is further underlined by the constant striving of many online stores toward the lowest possible prices, which can be explained by the agent experiencing the most net gain when it spends as little currency as possible, i.e., by minimizing loss. In contrast, agents commonly spend more currency than theoretically necessary by acquiring luxury products, a phenomenon that can be explained by the registered gain of acquiring an object of high quality outweighing the loss of currency, i.e., by maximizing gain. Therefore, exchange-interactions present a spectrum: at one extreme, the limited gain due to poor product quality outweighs the loss of currency, as it is minimal. At the other extreme, the large gain perceived through the high quality of the product outweighs the large loss of currency, as registered gain is maximized.

However, the model we have examined is based on a non-conscious environment. The human component can be modeled by introducing altruistic resonance and evaluative observation into the picture. This resonance is often limited in physical exchange-interactions, as large monopolized stores only act as dissociated storefronts to the production process. Similarly, the agents responsible for selling the product are dissociated from the production process, effectively disconnecting them from the product. This directly affects the altruistic resonance as well as the registered quality of the product. By contrast, when examining instances where the agent registers a direct connection between the product and the agent selling it (i.e., art ateliers, niche stores, etc.), the agent registers the quality of the object as higher than if it were presented in a dissociated environment. This can be explained by coupling the selling agent’s gain with the quality of the product, as the selling agent registers the most success and therefore gain when customers are satisfied. Hence, the buying agent will register the product as higher-quality because the selling agent’s gain is directly coupled with the buying agent’s satisfaction, and therefore with the product quality. However, altruistic resonance also presents itself as an important factor. The greater the altruistic resonance with the person selling the product, the more the agent is willing to pay for it, as the evaluative gain–loss calculation is influenced by gain of the fifth dimension. Similarly, gain of the third dimension also plays an important role, specifically when discussing personal store environments that the agent frequents. Gain of the third dimension refers to relational gains, which in this case would be attained by acts such as tipping, which subsequently also yield gain of the fifth dimension. This points toward personal exchange-interactions tending to higher-quality products, as low-quality products can often be purchased with less loss of currency from other sources. Concurrently, a high-quality product allows for more registered gain through quality and consequently more potential value-enhancement through indirect gain-quality coupling.

Concluding this final excursion within the theoretical core, Kerdotism yields a structured sketch of buyer behavior in online-exchange-interactions as well as in the more complex setting of personal exchange-interactions. It predicts tendencies toward either high-quality/high-price or low-quality/low-price products, as well as tendencies toward higher quality and, on average, higher prices the agent is willing to pay in personal exchange interactions.

## Normativity and Evaluation: Target and Payoff

### Normativity

The aim of this section is to clarify and precisely define the kind of normativity the present framework targets. Primarily, the framework focuses on practical normativity, i.e., the reasoning as well as the guidance of action. This is, however, inevitably covalently linked with evaluative normativity, since evaluative registration supplies the representational inputs on which practical guidance operates (cf. the basal identifications in Section “The Basal Definitions”).

The framework does not provide objective moral normativity, i.e., it does not derive what is morally prohibited or forbidden in a mind-independent sense. Rather, it is constructed to allow for full subjectivity and individuality, and thereby models moral phenomena, where addressed, as emergent and revisable regularities in interaction. The concept of moral evaluation itself is not analyzed in this paper, but may be expanded upon in future work.

### Conceptual Clarification

This framework uses normativity in a reasons-first sense. Therefore, as discussed in Section “The Principle of Teleological Necessity”, an action can only be conceived with gain or avoidance of loss as the reason for the action. A reason is therefore one or more aspects of a situation that the agent epistemically registers and that render a certain action more profitable than either the current action or inaction, shifting the agent’s behavior accordingly (cf. the gain–loss format introduced in Section “The Basal Definitions” and disaggregated in Section “The Taxonomy of Gain”).

Since the model maintains full subjectivity of the agent, evaluation is strictly delimited by the agent’s epistemic registration (cf. Section “The Corporeal Substrate”), thereby allowing for prediction error. An action therefore need not necessarily yield gain or avert loss, but it must be conceived under these goals as registered by the agent.

Having constructed the evaluative architecture, it is vital to define when and how an agent will act (cf. Section “The Second Basal Postulate”). An agent will act based on the evaluation, as established alongside the action threshold, if the expected gain is greater than the expected loss and the gain overcomes the threshold of stochastic uncertainty. This constitutes the framework’s practical normativity: the agent should enact the conceived action once it passes these baselines. Inaction in the face of an action that would yield greater gain is only possible if the gain is uncertain, which would fall within the action threshold. Otherwise, the agent must act based on what it registers as the net-best.

The concept of value is directly linked with the concept of reason, as value itself is a shifting medium based on the principles of homeostasis and proximity (Sections “The Principle of Homeostasis”; “The Principle of Proximity”), as well as subjectivity. Accordingly, value is registered by the agent insofar as an entity presents itself as positive in gain with regard to the agent’s situation.

As a mechanism to maximize the precision of predictive output, the agent employs a feedback loop (successive evaluation), which shifts registration and weighting based on the difference between the expected and registered outcome of action (cf. Section “The Malleability of the Cognitive Architecture”). This serves the purpose of ensuring more accurate predictions in the future by minimizing the gap between the agent’s subjective predictions and objective reality.

### Evaluation

The evaluation described in this framework is based on the basal view that the agent is an isolated system only connected to its physical surroundings through epistemic registration (cf. Section “The Corporeal Substrate”). This registration is an aggregate of epistemic perception, the mere physical perception of stimuli, and subsequent epistemic transduction, i.e., the interpretation of said stimuli. This entails the core concept that the framework is built upon, namely subjectivity.

Accordingly, the model treats the consequences of an action as disaggregable into elementary substructures that yield either gain or loss (cf. Section “The Basal Definitions”). On this basis, the five dimensions of gain establish gain and loss as a commensurable medium between highly diverse inputs in the agent’s system (Section “The Taxonomy of Gain”). Throughout the evaluative process, elementary gain and loss are weighed in terms of expected magnitude and modulated by the principles of proximity and homeostasis (Sections “The Principle of Proximity”; “The Principle of Homeostasis”).

The resulting balance yields either a net gain or a net loss. If the agent expects net loss, it will not act; inaction here constitutes a valid evaluative output in the form of inhibitory control. If it predicts net gain, it should enact the conceived action insofar as the gain surpasses the post-evaluative threshold of stochastic uncertainty; if it does not surpass this threshold, the agent will reevaluate.

This yields three phases of evaluation: preemptive evaluation, concurrent evaluation (during action execution in light of new epistemic input), and successive evaluation (after action execution, once consequences are registered). Successive evaluation further functions as a feedback mechanism (Section “The Malleability of the Cognitive Architecture”): the gap between expected and registered outcomes recalibrates future registration and weighting, thereby reducing prediction error over time.

### Payoff

Kerdotism therefore presents a unified account of action theory anchored in entropy, covering reasons behind actions, the practical *should*, as well as value, and with it the dynamic feedback-loop through which a system changes. It attains the concept of constraint without an objective description of morals through subjectivity and interaction. At the same time, it avoids spiraling into arbitrariness by employing the principles of proximity and homeostasis, which, together with the feedback-loop, ensure relative evaluative stability. Another phenomenologically evident pattern emerging from Kerdotism is systemic divergence between agents, i.e., individuality. The concept of individuality is naturally emergent due to the deterministic nature of the model, which posits that the evaluative apparatus necessarily diverges as it adapts to the registration of the environment, which itself is subjective. The framework also accounts for incorrect evaluation as a consequence of subjective perception never perfectly depicting objective reality.

By extension, Kerdotism can be applied to higher-order phenomena as emerging from interaction between multiple evaluative systems that intend to minimize loss and maximize gain. This supports an understanding of such phenomena as aggregates of many subjective agents interacting with one another to find a steady state, rather than as an objective system that agents adhere to differently. In particular, it provides a route to model moral evaluation and possession as stabilized regularities in repeated interaction (cf. Section “The Derivation of Collective Behavior”), rather than as mind-independent facts. In this way, such macro-phenomena can be analyzed under a unified vocabulary and, via the commensurability of gain, can even be quantified to a degree. All in all, this framework and its description invite numerous objections, which will be addressed next.

## Objections and Replies

### Normativity, Hume’s Gap and Moore’s Fallacy

A natural objection is that the framework attempts to derive an *ought* from purely descriptive premises. The model, however, does not claim to yield a mind-independent moral *ought*. It targets a practical, agent-relative *should*: given the constitutive aim of systemic persistence (the negentropic drive; cf. Section “The Principle of Teleological Necessity”), an agent *should* enact the option it registers as yielding net gain over net loss, provided that expected gain exceeds the action threshold under stochastic uncertainty (cf. Section “The Second Basal Postulate”). Practical normativity is therefore constitutive of agency: to count as an acting agent at all is, on this account, to be responsive to reasons, here modeled in the gain–loss format. Under this conditional/constitutive reading, the model does not bridge Hume’s gap (Hume, 1739); it specifies what practical normativity amounts to for systems that count as agents within the present architecture, i.e., systems whose behavior is guided by gain–loss evaluation as modulated by proximity and homeostasis.

A related worry invokes Moore’s naturalistic fallacy (Moore, 1903): if *good* were simply identified with some natural property (such as gain), the theory would seem to reduce normativity to description. The framework avoids this by not offering *good simpliciter* as a collectively generalizable property, nor by claiming an analytic identity between *good* and any natural magnitude. Instead, *good* (and *bad*) are treated as agent-relative evaluative representations grounded in how a system registers its own and others’ states in the gain–loss format.

Since registration is irreducibly subjective and modulated by proximity and homeostasis, the model proposes no single natural property as the definition of *good* across agents; rather, it offers an explanatory account of how agents form and revise evaluative representations while preserving full agentic freedom of registration. Accordingly, the present use of *good*/*bad* is operational: it serves to track intra-agent evaluative representations rather than to provide a semantic analysis of evaluative predicates.

### Reductionism

A natural objection to a model such as Kerdotism is that reductionism might undermine its integrity. The framework does not reduce complex phenomena to an overly flattening metric; rather, it translates them into a format of evaluative representations. These representations retain the complexity of the original input via categorization, weighting, and the principles of proximity and homeostasis. This allows the model to fulfill its intended scope as an action-theoretical framework (cf. Sections “The Derivation of Collective Behavior” and “Case Study: Capitalism”). Since the scope of the framework is not negatively affected by these reductionist features, they do not pose an issue within the present model.

### Inter-Agent-Bridge

A related worry concerns the bridge from intra-agent dynamics to inter-agent regularities: it may be unclear how the successive evaluative feedback-loop is realized and stabilized within a social setting. Within the present framework, this bridge is provided by the fact that other agents constitute a concentrated and comparatively unpredictable source of prospective gain and prospective loss for systemic integrity.

Accordingly, social interaction can generate third- and fourth-dimensional loss (e.g., retaliation, exclusion, or reputational devaluation), which is registered and enters successive evaluation, thereby recalibrating the agent’s mapping over repeated interaction (cf. Section “The Derivation of Collective Behavior”). In this way, strategies that tend, in expectation, to yield maximal gain and minimal loss in social interaction are progressively stabilized at the population level, without requiring direct instruction or mind-independent moral facts.

The degree and shape of this stabilization depend on ordinary parameters of interaction. For instance, higher commensurability (lower epistemic opacity) increases the reliability with which other agents’ prospective loss is registered; stronger proximity attenuation reduces the weight of temporally distant reputational loss; and higher expected enforcement intensity (retaliation/exclusion costs) increases the weight of third- and fourth-dimensional loss. Conversely, high noise in registration and low interaction frequency can prevent convergence and instead yield fragmented or unstable local regularities.

Thus, the model does not imply that all agents assimilate identical understandings of social norms; rather, each agent reaches an individual steady state that remains dynamically adjustable as social interaction continues.

## Comparison with Leading Accounts

While the model proposes many aspects not seen in other accounts, it also incorporates elements that share similarities with preexisting approaches. The following section compares Kerdotism with its closest current analogues.

### Utilitarianism

Utilitarianism as proposed by Bentham (1789), Mill (1861) might at first glance appear similar to the present framework, insofar as both employ a weighting calculus oriented toward the maximization of a metric. The metrics, however, differ substantially, since the gain–loss format is not reduced to a single quality such as *happiness* or *well-being*. By introducing subjectivity, the gain–loss format attains applicability to any agent as an agent-relative metric. Additionally, it explicitly includes loss, enabling another dimension of behavioral modeling via loss aversion. While utilitarianism can accommodate loss in a broad sense, the isolated treatment of loss within Kerdotism yields greater analytical clarity. With respect to the weighting calculus, Kerdotism provides a more atomistic and case-specific architecture, whereas utilitarianism does not offer an analogous intra-agent description. Accordingly, utilitarianism is at best a superficial and largely verbal analogue to Kerdotism.

### Free-Energy-Principle

The Free-Energy Principle proposed by Friston (2010) is more conceptually similar to Kerdotism. In particular, it exhibits an indirect entropic anchor, as discussed in Section “Related Approaches and Outstanding Gaps”. Its central postulate is that agents orient their behavior toward minimizing variational free energy, a quantity that tracks uncertainty-related constraints on perception and action. In this sense, it is structurally analogous to the principle of proximity in Kerdotism, which acts to minimize stochastic interference with gain. This analogue is, however, partial, since the Free-Energy Principle proposes no equivalent to the principles of homeostasis and subjectivity, nor to the gain–loss format. Similarly, its relation to the principle of teleological necessity is only indirect via the entropic anchor. The negentropic drive described in Kerdotism further differs from its Free-Energy counterpart by treating negentropic success as action guidance rather than as the minimization of variational free energy. Accordingly, whereas the Free-Energy Principle offers a descriptive approach to systemic stabilization, Kerdotism proposes a practical and agent-relative evaluative architecture. While stochastic uncertainty plays a central role in both, they differ in how it is established and incorporated within the conceptual frame, even where this yields partially similar outcomes.

Turning to higher-order multi-agent settings, the two models diverge further. While active inference within the Free-Energy Principle remains a current topic of discussion (Parr et al., 2022), Kerdotism provides, within the present paper, an explicit route to concepts such as morals, possession, and capitalism. In this sense, Kerdotism offers a more vertically integrated account of action-theory, especially with respect to the more emergent parts of the model.

Even though both models are based on very similar grounds, the Free-Energy Principle focuses strictly on the minimization of variational free energy and does not describe a clear path toward higher-order normativity. Kerdotism incorporates the same entropic basis and concern with stochasticity, but expands this point of departure by introducing an independent metric and scaling factors, as well as an explicit evaluative architecture, its feedback-loop, and the resulting higher-order phenomena. Therefore, despite key differences and Kerdotism’s greater vertical and horizontal integration, the Free-Energy Principle remains a closer analogue than Utilitarianism.

### Teleosemantics

Teleosemantics, associated with Millikan (1984), Neander (1991), offers a naturalistic account of representational normativity by grounding correctness in proper function, fixed by selection history (Wright, 1973). In this respect it shares with Kerdotism the ambition to avoid sui generis moral primitives. However, teleosemantics primarily targets correctness conditions for representation (and misrepresentation) rather than an explicit intra-agent weighing architecture: it does not provide a commensurable decision metric for integrating heterogeneous considerations in deliberation. Kerdotism, by contrast, is designed to model practical normativity via an agent-relative gain–loss format, modulated by proximity and homeostasis, and thereby to connect persistence constraints to concrete choice and to higher-order social phenomena; the overlap between the approaches is therefore largely limited to their shared naturalistic ambition.

### Reinforcement Learning/Decision-Theory

Reinforcement Learning and decision-theoretic approaches model action selection as the optimization of a formal objective, typically by maximizing expected utility or expected cumulative reward under uncertainty (Savage, 1954; Sutton & Barto, 2018). In its canonical sequential form, this optimization is framed in terms of dynamic programming and Bellman-style optimality over state transitions (Bellman, 1957). This exhibits clear similarities with Kerdotism, especially in its emphasis on action as the maximization of a guiding metric; in that respect, Bellman-style recursion is broadly analogous to the kerdotistic picture of sequentially structured evaluation, though it should be distinguished from the learning-like feedback loop of successive evaluation. Yet, these approaches do not provide an explicit intra-agent evaluative architecture of the kind developed here. They do, however, supply a commensurable internal metric (utility/reward) that is structurally similar to the gain–loss format, while typically abstracting away from the role of subjectivity. Additionally, reinforcement-learning models make explicit the exploration–exploitation trade-off, which is not stated as such in Kerdotism but is implicitly necessitated by the framework’s evaluative dynamics. Reinforcement learning and decision-theoretic approaches therefore offer close analogues to some parts of the kerdotistic framework, while lacking its intended vertical integration.

### Contractarian/Game-Theoretic Accounts of Norms

Contractarian and game-theoretic accounts treat norms as solutions to social coordination and conflict under strategic interaction: norms are stabilized when they support mutually advantageous cooperation (or rule out costly conflict) given shared expectations about others’ behavior (Hobbes, 1651; Lewis, 1969). In formal terms, such stabilization is often characterized in equilibrium terms, where compliance is rational given others’ compliance (or given enforcement incentives) (Nash, 1950). Contemporary work develops this picture in evolutionary and repeated-game settings, showing how strategies can converge to stable conventions under iteration and selection (Axelrod, 1984; Bicchieri, 2006; Skyrms, 1996). Like the higher-order parts of Kerdotism, these approaches offer a powerful account of inter-agent stabilization and enforcement; yet they typically leave implicit the intra-agent evaluative architecture by which strategic considerations are registered, rendered commensurable, and weighed under subjectivity. Accordingly, contractarian and game-theoretic accounts provide close analogues to the collective stabilization mechanisms emphasized in Kerdotism, while lacking its intended vertical integration and explicit agent-level evaluative calculus.

## Methodological Approach

This article is a piece of conceptual theory construction. The framework was developed by (I) fixing a minimal empirical anchor on agency in terms of persistence under entropic pressure, (II) extracting a commensurable intra-agent evaluative format from phenomenological constraints on deliberation (the gain–loss scheme), and (III) specifying explicit axioms and postulates that jointly determine action selection under subjectively registered information.

Methodologically, the construction functions as an explication and unification project: it makes explicit a set of commitments and constraints that allow heterogeneous evaluative considerations to be represented within a single agent-level architecture, and it treats continuity across levels of description as an adequacy condition rather than as a conclusion reached by reduction. The explication is assessed by whether it (I) preserves the target explanatory roles (action-guidance, trade-off integration, and cross-scale continuity), (II) increases determinacy by fixing explicit internal variables and update principles (proximity, homeostasis, threshold, and feedback), and (III) yields comparative leverage against nearby descriptions that either rely on proxy objectives without an explicit intra-agent weighing procedure or leave commensurability implicit.

The construction proceeded iteratively. First, we introduced the Principle of Teleological Necessity as a constitutive constraint on what counts as action within the target domain. Second, we provided stipulative basal identifications of *good*/*bad* with perceived gain/loss and disaggregated gain into five axiological dimensions to represent heterogeneous evaluative inputs in a single internal medium. Third, we added independent weighting principles (proximity and homeostasis) and a threshold condition to make the deliberative procedure operationally explicit. Finally, we extended the intra-agent architecture to multi-agent settings to show how stable higher-order regularities (e.g., morals, possession, and exchange-mediated phenomena) can be modeled as population-level stabilizations of gain–loss-guided strategies under repeated interaction.

Throughout, the guiding adequacy criterion was explanatory continuity across levels of description: later derivations were required to remain constrained by, and interpretable in terms of, the earlier axioms and the commensurable gain–loss format. Points of contact and partial analogies with existing accounts (e.g., utilitarian and free-energy-based approaches) are treated as comparative benchmarks rather than as premises.

Although the present paper is conceptual, the architecture yields empirically assessable expectations. In particular, it predicts (I) asymmetric recalibration rates for negative vs. positive prediction error, (II) proximity-style attenuation of temporally distant or low-probability consequences, (III) homeostatic scaling of registered value as a function of need-states and scarcity, (IV) systematic effects of epistemic opacity/commensurability on social weighting, and (V) threshold-like patterns of inaction under high stochastic uncertainty. Concretely, the model predicts stronger recalibration following registered loss than following registered gain, and it predicts systematic attenuation of altruistic weighting under increased epistemic opacity in social interaction.

## Conclusion

Kerdotism proposes an agent-relative, vertically integrated account of evaluative architecture that connects physical primitives to higher-order normative behavior. Starting from the principle of teleological necessity as a constitutive constraint on action, the framework introduces a commensurable gain–loss format in which anticipated consequences are disaggregated into elementary aspects, categorized across five dimensions, and weighted by proximity and homeostasis to yield action-guiding evaluation. By incorporating an intra-agent feedback-loop, the account also captures adaptive recalibration over time and thereby provides a route from individual deliberation to the stabilization of morals and possession in repeated interaction.

The result is a unified modeling vocabulary for tracking cross-domain trade-offs within agents and for relating those trade-offs to population-level regularities in social settings. The central claim is not merely that agents pursue persistence, but that deliberation can be modeled as a structured gain–loss integration problem whose weighting dynamics constrain the stabilization of norm-like regularities in interaction.

Relative to leading naturalistic proposals, the distinctive contribution lies in making the intra-agent weighing procedure itself explicit and commensurable. Where other approaches often locate explanatory weight in proxy objectives, relational dynamics, or etiological function, Kerdotism treats the gain–loss calculus as the pivot that links subjective registration to choice and, in aggregate, to shifts in collective equilibria.

The model does not deliver first-order moral truths; rather, it treats morals as inter-agent regularities that are stabilized through repeated interaction and underwritten by subjectively evaluating agents. Accordingly, it does not posit objective moral facts within the framework. Moreover, the present paper offers a conceptual architecture rather than a strict formalism that would permit direct implementation.

Future work can expand the framework’s coverage by extending the analysis to additional behavioral patterns and by developing a more explicit formalization of the gain–loss calculus and its weighting dynamics, thereby enabling closer contact with computational and empirical modeling. More broadly, Kerdotism is offered as a constraint on how accounts of persistence, deliberation, and normativity can be connected within a single agent-level architecture.

## Data Availability

No datasets were generated or analysed during the current study.

## Footnotes

1 The concept of negentropic success/failure within this framework is distinct from concepts such as variational free energy (Friston, 2010), as it is not directly linked to entropic minimization, but instead only indirectly connected via the gain–loss-format

2 Conceptually analogous to personification, but broadened to cases where agency or intentional stance is attributed to a non-conscious entity

3 This does not mean that the acquired object is of greater value to the agent, but instead that the gain of acquiring the object outweighs the loss of currency at the moment of purchase

## Declarations

### AI usage

AI-based tools were used for language feedback and to improve the clarity and precision of the manuscript text. All content, arguments, interpretations, and conclusions were developed by the author, who takes full responsibility for the final version.
